# Anti-CCP-positive patients with RA have a higher 10-year probability of fracture evaluated by FRAX®: a registry study of RA with osteoporosis/fracture

**DOI:** 10.1186/s13075-018-1515-1

**Published:** 2018-01-30

**Authors:** Tien-Tsai Cheng, Shan-Fu Yu, Fu-Mei Su, Yin-Chou Chen, Ben Yu-Jih Su, Wen-Chan Chiu, Chung-Yuan Hsu, Jia-Feng Chen, Chi-Hua Ko, Han-Ming Lai

**Affiliations:** 1 Kaohsiung Chang Gung Memorial Hospital, No. 123, Dapi Road, Niao-Song District, Kaohsiung, Taiwan, Republic of China; 2 Chang Gung University College of Medicine, Kaohsiung, Taiwan

**Keywords:** Rheumatoid arthritis, Anti-cyclic citrullinated peptide, FRAX

## Abstract

**Background:**

Positive anticyclic citrullinated peptide (anti-CCP+) is associated with bone loss in patients with rheumatoid arthritis (RA). However, whether overall positivity or specific levels of anti-CCP are associated with prevalent fracture or a 10-year probability of fracture remains unclear.

**Methods:**

This interim analysis of an RA registry was conducted at Chang Gung Memorial Hospital in Kaohsiung (CGMHK) for RA-related osteoporosis/fracture. Consecutive patients with RA who had visited the rheumatology clinic at CGMHK since September 1, 2014, and fulfilled the classification criteria of RA were enrolled. The demographics, disease duration, Disease activity in 28 joints based on erythrocyte sedimentation rate (DAS28-ESR), lifestyle, evidence of previous fracture, risk factors of fracture in the Fracture Risk Assessment Tool (FRAX®), and FRAX® score of each participant were collected. Anti-CCP, rheumatoid factor (RF), erythrocyte sedimentation rate (ESR), C-reactive protein (CRP), and bone mineral density (BMD) were measured at enrollment. The patients were grouped by positivity or quartiles of anti-CCP level (I–IV).

**Results:**

Five hundred twenty-one patients with RA were enrolled through May 31, 2016. In total, 359 (68.9%) patients were anti-CCP+. Compared with anti-CCP− patients, anti-CCP+ patients had a significantly higher DAS28-ESR (*p* = 0.0001) and 10-year probability of major (15.0 [18.9] vs. 12.0 [15.3], *p* = 0.0461) or hip (5.0 [9.2] vs. 3.6 [8.2], *p* = 0.0118) fracture, but a significantly lower BMD of the FN (*p* = 0.0196). The rates of osteoporosis and previous fracture were comparable. There were 130, 127, 132, and 132 patients in groups I–IV, respectively. The DAS28-ESR was significantly different (*p* = 0.0001) among the groups and correlated to anti-CCP levels. The BMD and 10-year probability of major (*p* = 0.0067) and hip (*p* = 0.0013) fracture among the groups were also different.

**Conclusions:**

Anti-CCP+ RA patients had a higher 10-year probability of major or hip fracture, independent of anti-CCP levels, and a lower BMD of the FN than anti-CCP− patients.

## Background

Osteoporosis and related fragility fracture [1–3] are well-known complications of RA. The chronic inflammation [4], immobilization/disability, medication [5], and specific genetic background [6] of RA had been postulated as contributing factors.

The anticyclic citrullinated peptide (anti-CCP) test first became commercially available in 2002. Subsequent to its application, the presence of anti-CCP in RA patients was associated with both progressive joint destruction [7] and comorbidities [8]. Once available, it became an RA diagnostic tool to help clinicians identify patients with RA with a poor prognosis [9, 10].

The 1994 World Health Organization operational definition of osteoporosis is a postmenopausal BMD ≥ 2.5 SD below the mean value for healthy young women. As BMD decreases, the relative risk for fracture increases. Thus, low BMD is an effective means of identifying individuals at risk of fracture. However, several studies have illustrated that osteoporosis is not the sole factor for predicting fragility fracture [11, 12]. Measures other than BMD can identify individuals at a high fracture risk. In 2008, the Fracture Risk Assessment Tool (FRAX®) (https://www.sheffield.ac.uk/FRAX) was developed to assess the 10-year probability of fracture.

Although anti-CCP+ status is associated with low BMD in patients with RA [13], whether anti-CCP positivity is associated with previous fracture or a 10-year probability of fracture calculated by FRAX® in RA patients has not been delineated. In the present investigation, we aimed to explore the relationships between anti-CCP, previous fracture, and FRAX-based 10-year probability of fracture in established RA patients.

## Methods

### Study population and design

This interim analysis of RA-related osteoporosis/fracture in an RA registry was conducted at CGMHK. In the registry, consecutive RA patients who had visited the rheumatology clinic at CGMHK since September 1, 2014, and fulfilled the 1987 American College of Rheumatology (ACR) revised criteria [14] or the 2010 ACR/European League Against Rheumatism classification criteria for RA [9] were enrolled. Those who were < 20 years of age, had any malignancy within the past 5 years, had an estimated life expectancy < 3 years, or were unwilling to participate in the study were excluded from the registry program.

Clinical assessments included demographic data (age, height, weight, body mass index [BMI]), presence and/or levels of anti-CCP, and RF. Disease duration was defined as the time that elapsed between the onset of first disease-related symptoms and enrollment. RA activity was quantified by using a health assessment questionnaire and disease activity score was assessed using the Disease Activity Score in 28 joints based on ESR  (DAS28-ESR). Information on current medications at the time of registration, including disease-modifying antirheumatic drugs (DMARDs), antiosteoporosis regimens, glucocorticoids, and biologic disease-modifying antirheumatic drugs (bDMARDs), was collected. In addition, lifestyle, evidence of previous fragility fracture (history or radiographic), and risk factors for fragility fracture based on the FRAX® tool were recorded. The 10-year probability of major and hip fractures, calculated using the FRAX® tool with FN BMD (Taiwan version), of each patient was collected as well. Anti-CCP, ESR, CRP, and BMD were measured at enrollment using a dual-energy X-ray absorptiometry scanner (Delphi A; Hologic Corp., Waltham, MA, USA) for hip (total), FN, and lumbar spine (L1–L4) areas. All procedures were performed by International Society for Clinical Densitometry-certified technicians in accordance with the manufacturer’s standardized procedures. Anteroposterior radiographs of the thoracolumbar spine as well as kidney, ureter, and bladder x-rays were obtained at enrollment for each participant to evaluate any radiographic spinal fractures.

The participants were categorized into two groups according to anti-CCP-positive (anti-CCP+) or anti-CCP− status and into four groups (I–IV) according to anti-CCP level quartiles. The participants provided written informed consent and the Regional Ethical Review Board of CGMHK approved the study (104-3530B), which was performed according to the principles of the Helsinki declaration.

### Laboratory measurements

RF and anti-CCP were determined by immunonephelometry using a BN ProSpec System (Siemens Healthcare, Erlangen, Germany) as well as a second-generation Phadia ImmunoCAP 250 EliA CCP assay (Phadia, Freiburg, Germany) according to the manufacturers’ recommendations. A positive result for anti-CCP was above a cutoff value of 7 U/ml, whereas that for RF was a value > 15 IU/ml. CRP and ESR were measured by using nephelometric and Westergren methods, respectively.

### Statistical analyses

Data were checked for normality. Anti-CCP values were used as continuous variables and additionally categorized into quartiles. If the baseline characteristics had a skewed distribution, nonparametric methods were used for analysis. Independent groups were compared using the Mann-Whitney *U* test for continuous variables and the chi-square test for dichotomous variables. For comparing characteristics between anti-CCP quartiles, we used analysis of variance or the Kruskal-Wallis *H* test for comparing means or medians, respectively. Normally distributed continuous variables are expressed as mean ± SD, and nonnormally distributed variables are expressed as median (IQR). All analyses were performed using R version 3.1.2 software (R Foundation for Statistical Computing, Vienna, Austria). The level of significance was set at 0.05.

## Results

### Demographic and clinical characteristics

Table 1 shows the demographic and clinical characteristics of the 521 patients. A total of 359 (68.9%) patients were anti-CCP+. The median (IQR) age was 59 (14) years, BMI was 23.1 (5) kg/m^2^, and disease duration was 11 (13) years. The study population consisted mainly of women (*n* = 447; 85.8%), of whom 80.3% were postmenopausal. The median (IQR) menopause age was 50 (2) years.

**Table 1:** Participants’ demographics and clinical characteristics

	All	Anti-CCP+ (≥7 U/ml)	Anti-CCP− (<7 U/ml)	*p* Value
No. of subjects (%)^a^	521 (100)	359 (68.9)	162 (31.1)	
Female sex, *n* (%)^a^	447 (85.8)	306 (85.24)	141 (87.04)	0.5858
Menopause, *n* (%)^a^	359 (80.3)	252 (82.4)	107 (75.9)	0.1101
Menopause, years^b^	50.0 (2.0)	50.0 (2.0)	50.0 (2.0)	0.6553
Age, years^b^	59.0 (14.0)	60.0 (14.0)	58.0 (14.0)	0.1700
BMI,^b^ kg/m^2^	23.1 (5.0)	23.0 (5.1)	23.1 (4.3)	0.7470
Symptom to diagnosis, years^b^	2.0 (6.0) *n* = 508	2.0 (6.0) *n* = 159	2.0 (6.0) *n* = 349	0.4732
Disease duration, years^b^	11.0 (13.0) *n* = 491	12.0 (13.0) *n* = 335	10.5 (13.5) *n* = 156	0.6656
Comorbidity, *n* (%)^a^	315 (60.5)	213 (59.3)	102 (63.0)	0.4326
Anti-CCP, U/ml, mean (SD)^b^	62.0 (294.9)	183.0 (283.0)	0.9 (1.1)	<0.0001
RF+, *n* (%)^a^	347 (67.1) *n* = 517	306 (86.2) *n* = 355	41 (25.3) *n* = 162	<0.0001
RF, IU/ml^b^	56.6 (189.4) *n* = 517	129.0 (288.9) *n* = 355	10.6 (6.8) *n* = 162	<0.0001
ESR, mm/h ^b^	17.0 (22.0)	19.0 (24.0)	13.5 (18.0)	<0.0001
CRP, mg/L ^b^	2.5 (7.7) *n* = 519	3.2 (8.6) *n* = 358	1.7 (5.6) *n* = 161	0.0005
DAS28-ESR^b^	3.1 (1.7) *n* = 519	3.2 (1.7) *n* = 358	2.8 (1.4) *n* = 161	0.0001
bDMARD, *n* (%)^a^	94 (18.0)	71 (19.8)	23 (14.2)	0.1253
Glucocorticoid users, *n* (%)^a^	452 (86.8)	317 (88.3)	135 (83.3)	0.1215
Alcohol ≥ 3 U/day, *n* (%)^a^	6 (1.2)	4 (1.1)	2 (1.2)	0.9051
Current smoker, *n* (%)^a^	35 (6.7)	30 (8.4)	5 (3.1)	0.0261
Fall in the previous year, *n* (%)^a^	97 (19.1) *n* = 507	69 (19.8) *n* = 348	28 (17.6) *n* = 159	0.5559
Parent fractured hip, *n* (%)^a^	37 (7.1)	25 (7.0)	12 (7.6)	0.8552
BMD, g/cm^2^				
Spine (L1–L4)^b^	0.858 (0.209) *n* = 501	0.850 (0.210) *n* = 343	0.883 (0.187) *n* = 158	0.0674
Hip (total)^c^	0.780 ± 0.146 *n* = 497	0.774 ± 0.145 *n* = 342	0.794 ± 0.149 *n* = 155	0.1518
FN^b^	0.620 (0.147) *n* = 497	0.614 (0.144) *n* = 342	0.643 (0.142) *n* = 155	0.0196
Osteoporosis,^d^ *n* (%)^a^	150 (29.47) *n* = 509	111 (31.7) *n* = 350	39 (24.5) *n* = 159	0.0993
Previous fracture, *n* (%)^a^	97 (18.6)	67 (18.7)	30 (18.5)	0.9687
Current antiosteoporosis, *n* (%)^a^	104 (20.0)	27 (16. 7)	77 (21.5)	0.2062
Major^b,e^	14.0 (17.2)	15.0 (18.9)	12.0 (15.3)	0.0461
Hip^b,f^	4.5 (8.5)	5.0 (9.2)	3.6 (8.2)	0.0118

*Abbreviations: BMI* Body mass index, *Anti-CCP* Anticitrullinated protein antibodies, *RF* Rheumatoid factor, *ESR* Erythrocyte sedimentation rate, *CRP* C-reactive protein, *DAS28-ESR* Disease Activity Score in 28 joints based on erythrocyte sedimentation rate, *bDMARD* Biologic disease-modifying antirheumatic drug, *BMD* Bone mineral density, *FN* Femoral neck

^a^ Absolute number (percent)

^b^ Median (IQR)

^c^ Mean ± SD

^d^ FN T-score less than or equal to −2.5

^e^ Ten-year probability of major fracture

^f^ Ten-year probability of hip fracture

Compared with anti-CCP− patients, anti-CCP+ patients had significantly higher levels of anti-CCP (U/ml) (183 [283] vs. 0.9 [1.1], *p* < 0.0001), RF (IU/ml) (129 [288.9] vs. 10.6 [6.8], *p* < 0.0001), ESR (mm/h) (19 [24] vs. 13.5 [18.0], *p* < 0.0001), CRP (mg/L) (3.2 [8.6] vs. 1.7 [5.7], *p* = 0.0005), DAS28-ESR (3.2 [1.7] vs. 2.8 [1.4], *p* = 0.0001), and higher RF+ rate (86.2% vs. 25.3%, *p* < 0.0001). The two groups were comparable in terms of demographics, rate of antiosteoporosis regimens, glucocorticoids and bDMARD, and comorbidities.

### Clinical characteristics pertinent to osteoporosis and fracture in the study population

**Fig. 1:**
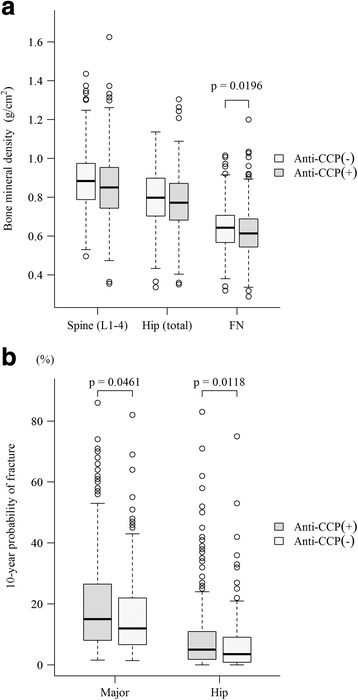
Association between positivity of anti-citrullinated protein antibodies (anticyclic citrullinated peptide-positive [anti-CCP+] or anti-CCP−) and systemic bone mineral density (**a**) or 10-year probability of fracture (**b**) in RA patients. *FN* Femoral neck

The prevalence of osteoporosis (FN; T-score less than or equal to −2.5) and rate of prevalent fracture were comparable between the anti-CCP+ and anti-CCP− groups (31.7% vs. 24.5%, *p* = 0.0993; and 18.7% vs. 18.5%, *p* = 0.9687). Compared with anti-CCP− patients, anti-CCP+ patients had a significantly lower FN BMD (g/cm^2^) level (0.614 [0.144] vs. 0.643 [0.142], *p* = 0.0196) (Fig. 1a), as well as a significantly higher smoking rate (8.4% vs. 3.1%, *p* = 0.0261). In terms of 10-year probability of major and hip fractures defined using the FRAX® tool, anti-CCP+ patients demonstrated a significantly higher probability than anti-CCP− patients (15.0 [18.9] vs. 12.0 [15.3, *p* = 0.0461) and (5.0 [9.2] vs. 3.6 [8.2], *p* = 0.0118), respectively (Table 1, Fig. 1b).

### Clinical characteristics of the four subgroups (anti-CCP level quartiles)

There were 130, 127, 132, and 132 patients in groups I–IV, respectively (Table 2). Age, sex, rate of menopause, age at menopause, BMI, time from symptoms to diagnosis, disease duration, rate of antiosteoporosis regimens/glucocorticoids/bDMARDs, and comorbidities were balanced between the groups, whereas the smoking rate (*p* = 0.0075) was significantly different among groups (Table 2).

**Table 2:** Participants’ clinical characteristics according to anticyclic citrullinated peptide quartile

	Group (anti-CCP U/ml)				
	I ≤2 (Q1) (*n* = 130)	II >2–60 (Q2) (*n* = 127)	III >60–290 (Q3) (*n* = 132)	IV ≥290 (Q4) (*n* = 132)	*p* Value
Anti-CCP, U/ml^a^	0.7 (0.7)	20.0 (35.2)	140.0 (108.0)	340.0 (34.0)	<0.0001
Female, *n* (%)^a^	114 (87.7)	111 (87.4)	112 (84.9)	110 (83.3)	0.2529
Age, years^b^	57.0 (14.0)	60.0 (14.0)	60.0 (13.5)	61.0 (14.0)	0.0729
BMI, kg/m^2b^	23.3 (4.2)	22.6 (5.0)	23.0 (5.4)	23.8 (5.1)	0.3731
Disease duration, years^b^	11.0 (13.0) *n* = 126	12.0 (13.0) *n* = 121	12.0 (15.0) *n* = 121	12.0 (11.0) *n* = 123	0.8547
Symptom to diagnosis, years^b^	10.0 (8.5) *n* = 127	10.0 (8.0) *n* = 127	9.0 (10.5) *n* = 132	10.0 (11.0) *n* = 131	0.9379
DAS28-ESR^b^	2.7 (1.4) *n* = 129	3.1 (1.6)	3.0 (1.6) *n* = 131	3.6 (2.0)	0.0001
RF+, *n* (%)^a^	22 (16.9)	95 (74.8)	111 (86.1) *n* = 129	119 (90.8) *n* = 131	<0.0001
RF, IU/ml^b^	10.3 (1.3)	88.8 (205.5)	125.0 (256.3) *n* = 129	146.0 (469.0) *n* = 131	<0.0001
ESR, mm/h^b^	12.5 (14.0)	18.0 (26.0)	17.0 (20.0)	23.0 (34.0)	<0.0001
CRP, mg/L^b^	1.4 (5.7) *n* = 129	3.1 (8.2)	3.1 (8.6)	3.2 (9.2) *n* = 131	0.0012
Current smoking, *n* (%)^a^	4 (3.1)	6 (4.7)	11 (8.3)	14 (10.6)	0.0075
Glucocorticoid users, *n* (%)^a^	107 (82.3)	113 (89.0)	117 (88.6)	115 (87.1)	0.2876
Comorbidity, *n* (%)^a^	80 (61.5)	83 (65.4)	75 (56.8)	77 (58.3)	0.3448
Fall in previous year, *n* (%)^a^	22 (17.3) *n* = 127	26 (20.6) *n* = 126	28 (22.1) *n* = 127	21 (16.5) *n* = 127	0.9521
BMD, g/cm^2^					
Spine (L1–L4)^a^	0.893 (0.195) *n* = 128	0.826 (0.211) *n* = 123	0.854 (0.223) *n* = 122	0.856 (0.180) *n* = 128	0.0394
Hip (total)^c^	0.807 ± 0.148 *n* = 124	0.741 ± 0.122 *n* = 122	0.786 ± 0.148 *n* = 125	0.786 ± 0.157 *n* = 126	0.0041
FN^b^	0.651 (0.149) *n* = 124	0.602 (0.146) *n* = 122	0.615 (0.145) *n* = 125	0.6145 (0.139) *n* = 126	0.0033
Previous fracture, *n* (%)^a^	20 (15.4)	28 (22.1)	23 (17.4)	26 (19.7)	0.5827
Osteoporosis,^a,d^ *n* (%)	27 (21.1) *n* = 128	45 (36.0) *n* = 125	38 (29.9) *n* = 127	40 (31.0) *n* = 129	0.1866
Current anti-osteoporosis, *n* (%)^a^	18 (13.9)	34 (26.8)	25 (19.0)	27 (20.6)	0.4225
Major^b,e^	11.0 (13.2)	17.0 (19.6)	15.0 (17.4)	14.0 (19.7)	0.0067
Hip^b,f^	3.1 (6.3)	6.1 (9.8)	4.6 (9.0)	4.9 (9.4)	0.0013

*Abbreviations: BMI* Body mass index, *Anti-CCP* Anticitrullinated protein antibodies, *RF* Rheumatoid factor, *ESR* Erythrocyte sedimentation rate, *CRP* C-reactive protein, *DAS28-ESR* Disease Activity Score in 28 joints based on erythrocyte sedimentation rate, *BMD* Bone mineral density, *FN* Femoral neck

^a^ Absolute number (percentage)

^b^ Median (interquartile range)

^c^ Mean ± SD

^d^ FN T-score less than or equal to −2.5

^e^ Ten-year probability of major fracture

^f^ Ten-year probability of hip fracture

Regarding variables related to RA disease activity, the rate of RF+ (*p* < 0.0001) and levels of RF (*p* < 0.0001), ESR (*p* < 0.0001), CRP (*p* = 0.0012), and DAS28-ESR (*p* = 0.0001) were significantly different among the groups and correlated with anti-CCP levels. BMD at the spine (L1–L4) (*p* = 0.0394), hip (total) (*p* = 0.0041), and FN (*p* = 0.0033) differed significantly among the groups (Table 2), whereas the prevalence of osteoporosis, rate of previous fracture, and number of falls in the previous year were comparable. The 10-year probabilities of major fracture (*p* = 0.0067) and hip fracture (*p* = 0.0013) among the groups were significantly different (Table 2).

**Fig. 2:**
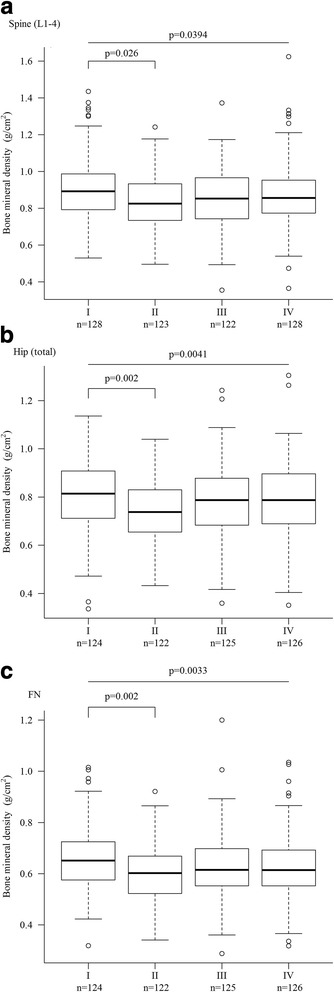
Bone mineral density at the spine (L1–L4) (**a**), hip (total) (**b**), and FN (**c**) in subgroups (I–IV) of RA patients stratified according to quartiles of anti-CCP levels. *FN* femoral neck

**Fig. 3:**
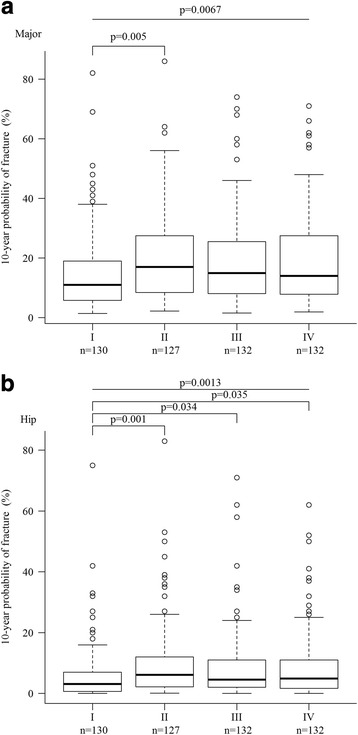
Ten-year probability of major (**a**) and hip (**b**) fracture in subgroups (I–IV) of RA patients stratified according to quartiles of anti-CCP levels

A subgroup analysis revealed that the BMD values at the spine (L1–L4) (*p* = 0.026), hip (total) (*p* = 0.002), and FN (*p* = 0.002) in group II, but not in group III or IV, were significantly lower than those in group I (Fig. 2a–c). In addition, there was a significantly higher 10-year probability of major fracture (*p* = 0.005) (Fig. 3a) in group II than in group I. The 10-year probabilities of hip fracture in group II (*p* = 0.001), III (*p* = 0.034), and IV (*p* = 0.035) (Fig. 3b) were significantly higher than that of group I.

## Discussion

Our investigation revealed that the anti-CCP levels were correlated with RA disease activity. Anti-CCP+ patients with RA had a significantly lower BMD level at the FN than anti-CCP− RA patients. The BMD at all sites was significantly different among groups I–IV, although the subgroup analysis revealed that the difference occurred only between groups I and II (Table 2, Fig. 1a). Neither anti-CCP positivity nor anti-CCP level was correlated with the prevalence of osteoporosis or of fracture among patients with RA. The 10-year probability of fracture, either major or hip, in anti-CCP+ RA patients was significantly higher than that in anti-CCP− RA patients, although the 10-year probability of fracture was not correlated with anti-CCP level.

Guler et al. first reported that anti-CCP+ RA patients had lower BMD than anti-CCP− RA patients at the FN but not in the spine [13]. In addition, Orsolini et al. illustrated that anti-CCP+ patients not only had a higher prevalence of osteopenia/osteoporosis but also had a negative titer-dependent effect on BMD at the FN but not at the lumbar spine in patients with established RA [15]. A recent publication by Wysham et al. also demonstrated that a high positive anti-CCP level and appendicular lean muscle mass were associated with FN BMD in established RA [16]. Llorente et al. reported that patients with early RA with anti-CCP+ had lower BMD than the anti-CCP− control subjects at the lumbar spine, FN, and hip [17]. Bugatti et al. also demonstrated that anti-CCP+ patients with early untreated RA had lower BMD than their anti-CCP− counterparts at the lumbar spine and hip [18]. Similar to the findings in studies on patients with established RA [13, 15, 16], the anti-CCP+ patients in our cohort had a significantly lower BMD than anti-CCP− patients at the FN but not at the lumbar spine or hip. However, we did not see a titer-dependent correlation between anti-CCP levels and BMD at all sites (Table 2).

Both previous studies [17, 18] pertaining to the association of anti-CCP and BMD in patients with early RA revealed a negative effect of anti-CCP+ on BMD at all measured sites, a finding that is inconsistent with those of the present study and other series on established RA [13, 15]. There are several possible explanations for the different observations between the studies. The first one is the differences in the enrolled populations in the aforementioned studies. All regimens, including glucocorticoids [5], conventional DMARDs [19], and bDMARDs [20, 21], used to treat RA revealed a BMD-modulating effect. After long-term treatment of patients with established RA, BMD may vary depending on which regimen or how long the regimens are used. In addition, the disease activity (DAS28-ESR) in the present series was 3.1 (1.7), which might differ from that of the Bugatti et al. study [18]. Because it has been postulated that higher disease activity predisposes patients with RA to bone loss [22], the difference in disease activity between the study populations may confer the divergent effect on the BMD. Regardless, it appears conceivable that anti-CCP+, especially when highly positive [15, 16], in either patients with early or established RA is associated with lower BMD, especially at the FN, than in their anti-CCP− counterparts.

The causes of the higher prevalence of osteoporosis in patients with long-term RA than in the general population are multifactorial. In addition, why anti-CCP positivity or levels are associated with low BMD in patients with RA is not fully understood. It seems most plausible that anti-CCP can mediate osteoclast differentiation and activity [23, 24] that may result in bony erosion and bone loss in patients with RA.

Similarly to osteoporosis, the causes of the higher prevalence of fragility fracture in patients with long-term RA than in the general population are multifactorial. The prevalence of RA-associated fragility fracture is reportedly 13–27% [3, 25, 26]. The rate of previous fracture, as defined by the FRAX® tool, in our series was 18.6%. In the present investigation, a heterogeneous and real-world RA population composed of men and women of different ages, serotypes, and disease activities was enrolled. In addition, both clinical and radiological morphometric vertebral fractures were included, unlike previous studies in which either vertebral fractures [26] or hip fractures [27] were counted. These discrepancies in the populations enrolled and the method of prevalent fracture calculation may explain the differences between series in the prevalence of fractures.

Mohammad et al. found that a longer duration of RA, markers of disease activity, and disease severity were risk factors for asymptomatic vertebral fractures [26]. In addition, they reported that anti-CCP+ status was an independent risk factor for asymptomatic vertebral fracture. Although we found that a higher anti-CCP level was associated with higher disease activity (DAS28-ESR) and higher acute-phase reactant levels (ESR, CRP) (Table 2) in our series, we did not see an association between anti-CCP positivity or levels and previous fractures. The present investigation and that of Mohammad et al. were cross-sectional studies. Whether anti-CCP is associated with future new incident fractures needs further investigation.

### Anti-CCP and 10-year probability of fracture

Although there are many fracture risk assessment tools available worldwide, the most widely used in Taiwan is the FRAX® tool (Taiwan version). Its use can help in estimation of the 10-year absolute risk of major and hip fractures among individuals ≥ 40 years old. The FRAX® tool includes parameters such as sex, age, BMI, previous fracture, smoking, glucocorticoid use, diagnosis of RA, and BMD. However, shortcomings exist for rheumatologists using the FRAX® tool to evaluate the risk of fracture specifically for RA patients, because the tool does not include other well-known risk factors of fracture for patients with RA, such as disease activity or duration. Despite its shortcomings, this tool has been validated in several countries, except in the United Kingdom [28], for RA patients [29, 30].

Using the FRAX® tool, we found that anti-CCP+ RA patients had a higher risk of 10-year probability of major or hip fracture than anti-CCP− RA patients (Table 1, Fig. 1b). In addition, anti-CCP+ RA patients had a higher 10-year probability of hip fracture than anti-CCP− RA patients at all anti-CCP levels (Table 2, Fig. 3b). The reason why anti-CCP+ is associated with an elevated 10-year probability of fracture in our cohort is twofold. First, in terms of risk factors in the FRAX® tool, we found the rates of smokers and FN BMD in anti-CCP+ patients were significantly higher than those of anti-CCP− patients. Second, other risk factors, including age, previous fracture, and glucocorticoid use, in anti-CCP+ patients were numerically, albeit not significantly, higher than in anti-CCP− patients (Table 1). In addition, we found that if the factors of FN BMD and smoking were excluded from the calculation of FRAX® score, anti-CCP+ RA patients still had significantly higher FRAX® scores (hip) (data not shown) than their anti-CCP− counterparts. This suggests that the summation effect of other risk factors can explain the differences in the FRAX® score (hip) between the two groups as well.

Regarding the relationship between anti-CCP level and fracture risk, only the risk of major fracture was higher in group II than in group I (*p* = 0.005) (Fig. 3a), whereas the risks of hip fracture were significantly higher in groups II–IV than in group I. The smoking rate was proportional to the anti-CCP levels (Table 2). However, BMD was not proportional to the anti-CCP levels. The elevated risk of major fracture in group II and the rates of hip fracture in groups II–IV can also been explained by the summation effect of other risk factors in the FRAX® tool. These findings suggest that, in the future, those using the FRAX® tool to evaluate the fracture risk for patients with RA should consider the positivity/titer of anti-CCP, because it has been suggested for adjustment of FRAX® score by doses of glucocorticoid [31].

The disease activity markers of ESR, CRP, and DAS28-ESR were significantly different between patients with anti-CCP+ and anti-CCP− status and among groups I–IV. The marker levels were proportional to the anti-CCP levels in our cohort. Whether anti-CCP-associated higher disease activity in patients with RA is related to the long-term risk of fracture requires further validation studies.

The present study has limitations. This investigation is a cross-sectional study of patients with established RA. Although reversion from anti-CCP+ to anti-CCP− status was not observed in a 2-year observational study [32], the anti-CCP levels are subject to treatment changes in patients with RA [33, 34]. Whether the positivity or levels of anti-CCP after long-term treatment (10 years) in our cohort were changed is unknown. However, because the anti-CCP+ rate (68.9%) of our cohort was compatible with that of a previous study [35], it seems that long-term treatment in our cohort would not change the positivity of anti-CCP. Hence, the application of anti-CCP in the prediction of 10-year probability of fracture in established RA appears reliable. In addition, the application of the FRAX® tool for estimating fracture risk is limited to populations aged ≥ 40 years. In addition, anti-CCP positivity, disease activity, and disease duration were not included in the FRAX® tool in the evaluation of 10-year probability of fracture for RA patients. Whether the application of anti-CCP positivity could be used to estimate the 10-year probability of fracture by using the FRAX® tool with younger patients with RA warrants further investigation. Finally, the heterogeneity of our cohort fulfilled the 1987 or 2010 ACR classification criteria, which could be considered another drawback of our study. Whether the sensitivity of using anti-CCP for estimating the 10-year probability of fracture for RA patients would be the same under different criteria is unknown.

## Conclusions

Anti-CCP+ RA patients had a lower FN BMD than anti-CCP− RA patients, and the BMD at the measured sites was not related to anti-CCP levels in RA patients. By using the FRAX® tool, we found that anti-CCP + RA patients had a higher 10-year probability of major or hip fracture independent of anti-CCP level. Studies designed to explore whether there is a difference in incident fractures between anti-CCP+ and anti-CCP− RA patients or whether anti-CCP should be incorporated into the updated version of the FRAX® tool for RA are needed to determine the real impact of our findings.

## Abbreviations

ACR: American College of Rheumatology
Anti-CCP: Anticyclic citrullinated peptide
bDMARD: Biologic disease-modifying antirheumatic drug
BMD: Bone mineral density
BMI: Body mass index
CGMHK: Chang Gung Memorial Hospital in Kaohsiung
CRP: C-reactive protein
DAS28-ESR: Disease Activity Score in 28 joints based on erythrocyte sedimentation rate
DMARD: Disease-modifying antirheumatic drug
ESR: Erythrocyte sedimentation rate
FN: Femoral neck
FRAX®: Fracture Risk Assessment Tool
RA: Rheumatoid arthritis
RF: Rheumatoid factor

## References

[1] Haugeberg G, Uhlig T, Falch JA, Halse JI, Kvien TK (2000). Reduced bone mineral density in male rheumatoid arthritis patients: frequencies and associations with demographic and disease variables in ninety-four patients in the Oslo County Rheumatoid Arthritis Register. Arthritis Rheum.

[2] Huusko TM, Korpela M, Karppi P, Avikainen V, Kautiainen H, Sulkava R (2001). Threefold increased risk of hip fractures with rheumatoid arthritis in central Finland. Ann Rheum Dis.

[3] Rentero ML, Amigo E, Chozas N, Fernández Prada M, Silva-Fernández L, Abad Hernandez MA (2015). Prevalence of fractures in women with rheumatoid arthritis and/or systemic lupus erythematosus on chronic glucocorticoid therapy. BMC Musculoskelet Disord.

[4] Haugeberg G, Conaghan PG, Quinn M, Emery P (2009). Bone loss in patients with active early rheumatoid arthritis: infliximab and methotrexate compared with methotrexate treatment alone. Explorative analysis from a 12-month randomised, double-blind, placebo-controlled study. Ann Rheum Dis.

[5] Saag KG (2014). Bone safety of low-dose glucocorticoids in rheumatic diseases. Ann NY Acad Sci.

[6] Mohamed RH, Mohamed RH, El-Shahawy EE (2016). Relationship between RANK and RANKL gene polymorphisms with osteoporosis in rheumatoid arthritis patients. Genet Test Mol Biomarkers.

[7] Syversen SW, Gaarder PI, Goll GL, Ødegård S, Haavardsholm EA, Mowinckel P (2008). High anti-cyclic citrullinated peptide levels and an algorithm of four variables predict radiographic progression in patients with rheumatoid arthritis: results from a 10-year longitudinal study. Ann Rheum Dis.

[8] Arnab B, Biswadip G, Arindam P, Shyamash M, Anirban G, Rajan P (2013). Anti-CCP antibody in patients with established rheumatoid arthritis: does it predict adverse cardiovascular profile?. J Cardiovasc Dis Res.

[9] Aletaha D, Neogi T, Silman AJ, Funovits J, Felson DT, Bingham CO (2010). 2010 rheumatoid arthritis classification criteria: an American College of Rheumatology/European League Against Rheumatism collaborative initiative. Arthritis Rheum.

[10] Hecht C, Englbrecht M, Rech J, Schmidt S, Araujo E, Engelke K (2015). Additive effect of anti-citrullinated protein antibodies and rheumatoid factor on bone erosions in patients with RA. Ann Rheum Dis.

[11] Kanis JA, Borgstrom F, De Laet C, Johansson H, Johnell O, Jonsson B (2005). Assessment of fracture risk. Osteoporos Int.

[12] Siris ES, Brenneman SK, Barrett-Connor E, Miller PD, Sajjan S, Berger ML (2006). The effect of age and bone mineral density on the absolute, excess, and relative risk of fracture in postmenopausal women aged 50–99: results from the national osteoporosis risk assessment (NORA). Osteoporos Int.

[13] Guler H, Turhanoglu AD, Ozer B, Ozer C, Balci A (2008). The relationship between anti-cyclic citrullinated peptide and bone mineral density and radiographic damage in patients with rheumatoid arthritis. Scand J Rheumatol.

[14] Arnett FC, Edworthy SM, Bloch DA, McShane DJ, Fries JF, Cooper NS (1988). The American Rheumatism Association 1987 revised criteria for the classification of rheumatoid arthritis. Arthritis Rheum.

[15] Orsolini G, Caimmi C, Viapiana O, Idolazzi L, Fracassi E, Gatti D (2017). Titer-dependent effect of anti-citrullinated protein antibodies on systemic bone mass in rheumatoid arthritis patients. Calcif Tissue Int.

[16] Wysham KD, Shoback DM, Imboden Jr JB, Katz PP. Association of high anti-cyclic citrullinated peptide seropositivity and lean mass index with low bone mineral density in rheumatoid arthritis. Arthritis Care Res (Hoboken); 2017. https://doi.org/10.1002/acr.23440. 10.1002/acr.23440 PMC5936473 29106028

[17] Llorente I, Merino L, Ortiz AM, Escolano E, González-Ortega S, García-Vicuña R (2017). Anti-citrullinated protein antibodies are associated with decreased bone mineral density: baseline data from a register of early arthritis patients. Rheumatol Int.

[18] Bugatti S, Bogliolo L, Vitolo B, Manzo A, Montecucco C, Caporali R (2016). Anti-citrullinated protein antibodies and high levels of rheumatoid factor are associated with systemic bone loss in patients with early untreated rheumatoid arthritis. Arthritis Res Ther.

[19] Zonneveld IM, Bakker WK, Dijkstra PF, Bos JD, van Soesbergen RM, Dinant HJ (1996). Methotrexate osteopathy in long-term, low-dose methotrexate treatment for psoriasis and rheumatoid arthritis. Arch Dermatol.

[20] Chen HA (2006). The effect of etanercept on anti-cyclic citrullinated peptide antibodies and rheumatoid factor in patients with rheumatoid arthritis. Ann Rheum Dis.

[21] Briot K, Rouanet S, Schaeverbeke T, Etchepare F, Gaudin P, Perdriger A (2015). The effect of tocilizumab on bone mineral density, serum levels of Dickkopf-1 and bone remodeling markers in patients with rheumatoid arthritis. Joint Bone Spine.

[22] Guler-Yuksel M, Allaart CF, Goekoop-Ruiterman YPM, de Vries-Bouwstra JK, van Groenendael JH, Mallée C (2009). Changes in hand and generalised bone mineral density in patients with recent-onset rheumatoid arthritis. Ann Rheum Dis.

[23] Harre U, Georgess D, Bang H, Bozec A, Axmann R, Ossipova E (2012). Induction of osteoclastogenesis and bone loss by human autoantibodies against citrullinated vimentin. J Clin Invest.

[24] Krishnamurthy A, Joshua V, Haj Hensvold A, Jin T, Sun M, Vivar N (2016). Identification of a novel chemokine-dependent molecular mechanism underlying rheumatoid arthritis-associated autoantibody-mediated bone loss. Ann Rheum Dis.

[25] van Staa TP, Geusens P, Bijlsma JW, Leufkens HG, Cooper C (2006). Clinical assessment of the long-term risk of fracture in patients with rheumatoid arthritis. Arthritis Rheum.

[26] Mohammad A, Lohan D, Bergin D, Mooney S, Newell J, O’Donnell M (2013). The prevalence of vertebral fracture on vertebral fracture assessment imaging in a large cohort of patients with rheumatoid arthritis. Rheumatology (Oxford).

[27] Yamamoto Y, Turkiewicz A, Wingstrand H, Englund M (2015). Fragility fractures in patients with rheumatoid arthritis and osteoarthritis compared with the general population. J Rheumatol.

[28] Klop C, de Vries F, Bijlsma JWJ, Leufkens HGM, Welsing PMJ (2016). Predicting the 10-year risk of hip and major osteoporotic fracture in rheumatoid arthritis and in the general population: an independent validation and update of UK FRAX without bone mineral density. Ann Rheum Dis.

[29] Lee JH, Suh YS, Koh JH, Jung SM, Lee JJ, Kwok SK (2014). The risk of osteoporotic fractures according to the FRAX model in Korean patients with rheumatoid arthritis. J Korean Med Sci.

[30] Friis-Holmberg T, Rubin KH, Brixen K, Tolstrup JS, Bech M (2014). Fracture risk prediction using phalangeal bone mineral density or FRAX®?—A Danish cohort study on men and women. J Clin Densitom.

[31] Kanis JA, Johansson H, Oden A, McCloskey EV (2011). Guidance for the adjustment of FRAX according to the dose of glucocorticoids. Osteoporos Int.

[32] Kastbom A, Forslind K, Ernestam S, Geborek P, Karlsson JA, Petersson IF (2016). Changes in the anticitrullinated peptide antibody response in relation to therapeutic outcome in early rheumatoid arthritis: results from the SWEFOT trial. Ann Rheum Dis.

[33] Cuchacovich M, Catalan D, Wainstein E, Gatica H, Soto L, Aravena O (2008). Basal anti-cyclic citrullinated peptide (anti-CCP) antibody levels and a decrease in anti-CCP titres are associated with clinical response to adalimumab in rheumatoid arthritis. Clin Exp Rheumatol.

[34] Iannone F, Tampoia M, Giannini M, Lopalco G, Cantarini L, Villalta CD (2016). Changes in anti-cyclic citrullinated peptide antibodies and rheumatoid factor isotypes serum levels in patients with rheumatoid arthritis following treatment with different biological drugs. Clin Exp Rheumatol.

[35] van Venrooij WJ, van Beers JJBC, Pruijn GJM (2011). Anti-CCP antibodies: the past, the present and the future. Nat Rev Rheumatol.

